# Fast and Slow Rhythms of Naturalistic Reading Revealed by Combined Eye-Tracking and Electroencephalography

**DOI:** 10.1523/JNEUROSCI.1849-22.2023

**Published:** 2023-06-14

**Authors:** Lena Henke, Ashley G. Lewis, Lars Meyer

**Affiliations:** ^1^Max Planck Institute for Human Cognitive and Brain Sciences, Research Group Language Cycles, Leipzig 04103, Germany; ^2^Neurobiology of Language Department, Max Planck Institute for Psycholinguistics, Nijmegen 6525 XD, The Netherlands; ^3^Donders Institute for Brain, Cognition and Behaviour, Radboud University, Nijmegen 6525 XD, The Netherlands; ^4^Clinic for Phoniatrics and Pedaudiology, University Hospital Münster, Münster 48149, Germany

**Keywords:** chunking, delta-band, eye movements, neural oscillations, reading, theta-band

## Abstract

Neural oscillations are thought to support speech and language processing. They may not only inherit acoustic rhythms, but might also impose endogenous rhythms onto processing. In support of this, we here report that human (both male and female) eye movements during naturalistic reading exhibit rhythmic patterns that show frequency-selective coherence with the EEG, in the absence of any stimulation rhythm. Periodicity was observed in two distinct frequency bands: First, word-locked saccades at 4-5 Hz display coherence with whole-head theta-band activity. Second, fixation durations fluctuate rhythmically at ∼1 Hz, in coherence with occipital delta-band activity. This latter effect was additionally phase-locked to sentence endings, suggesting a relationship with the formation of multi-word chunks. Together, eye movements during reading contain rhythmic patterns that occur in synchrony with oscillatory brain activity. This suggests that linguistic processing imposes preferred processing time scales onto reading, largely independent of actual physical rhythms in the stimulus.

**SIGNIFICANCE STATEMENT** The sampling, grouping, and transmission of information are supported by rhythmic brain activity, so-called neural oscillations. In addition to sampling external stimuli, such rhythms may also be endogenous, affecting processing from the inside out. In particular, endogenous rhythms may impose their pace onto language processing. Studying this is challenging because speech contains physical rhythms that mask endogenous activity. To overcome this challenge, we turned to naturalistic reading, where text does not require the reader to sample in a specific rhythm. We observed rhythmic patterns of eye movements that are synchronized to brain activity as recorded with EEG. This rhythmicity is not imposed by the external stimulus, which indicates that rhythmic brain activity may serve as a pacemaker for language processing.

## Introduction

Auditory neuroscience emphasizes the involvement of neural oscillations in speech and language (Meyer, 2018; Poeppel and Assaneo, 2020; Poeppel and Teng, 2020). Oscillations track acoustic rhythms to support speech perception and information uptake (Giraud and Poeppel, 2012; Poeppel and Assaneo, 2020). For example, theta-band oscillations (4-8 Hz) track syllables (Luo and Poeppel, 2007; Peelle et al., 2013; Doelling et al., 2014) and delta-band oscillations (<4 Hz) track prosodic phrases (Bourguignon et al., 2013; Molinaro et al., 2016). Tracking is also observed outside of audition: Occipital theta- and delta-band oscillations synchronize with speakers' lip movements (Crosse et al., 2015; Park et al., 2016; Bourguignon et al., 2020; Biau et al., 2021) and sign language (Brookshire et al., 2017).

Beyond stimulus tracking, oscillations serve endogenous functions, such as prediction and chunking (Giraud, 2020; Haegens, 2020; Kandylaki and Kotz, 2020; Klimovich-Gray and Molinaro, 2020; Lewis, 2020; Meyer et al., 2020a,b). For instance, theta-band oscillations guide the temporal prediction of speech (Bosker and Ghitza, 2018; Kösem et al., 2018, 2020) and delta-band oscillations subserve multi-word chunking (Ding et al., 2016; Meyer et al., 2017; Jin et al., 2020; Henke and Meyer, 2021). Supporting the possibly endogenous nature of chunking, delta-band phase can drive chunking even when diverging acoustic cues are present (Meyer et al., 2017; Henke and Meyer, 2021).

Studying endogenous functions is challenging when assessing speech because even subtle acoustic rhythms become confounds (Luo and Ding, 2020; Meyer et al., 2020a,b; Pinto et al., 2022). To overcome this problem, we here study simultaneous recordings of eye movements and EEG during naturalistic reading. Text itself does not impose temporal structure on the reader, as words occur in space rather than time. Thus, temporal behavioral or electrophysiological rhythms during reading would be endogenously imposed by the reader's brain. Accordingly, rhythmic fluctuations were reported for visuo-spatial attention shifting independent of external cues (Busch and VanRullen, 2010; Chakravarthi and VanRullen, 2012; Landau and Fries, 2012; Fiebelkorn et al., 2013; Dugué et al., 2015, 2016; Landau et al., 2015; McLelland et al., 2016). These appear to be tightly linked to saccadic eye movements, possibly optimizing visual response gain by increasing neuronal excitability for processing subsequent stimuli (Maldonado et al., 2008; Rajkai et al., 2008; Melloni et al., 2009; Ito et al., 2011; Hogendoorn, 2016). Rhythmic visual sampling even occurs independent of spatial eye movements (Re et al., 2019), suggesting that it provides an endogenous reference frame for information sampling.

We investigated whether eye movements during reading (i.e., saccades and fixations) exhibit temporal regularities that reflect the endogenous rhythms of oscillatory cycles in the brain. In reading, the eyes move from word to word every ∼200-250 ms (Rayner, 1998; Siegelman et al., 2022), leading to rhythmicity of saccades at 4-5 Hz (Gagl et al., 2021). While the corresponding electrophysiological frequency band (i.e., theta-band) tracks syllables in audition (Luo and Poeppel, 2007; Peelle et al., 2013; Doelling et al., 2014), its hypothetical role for sampling words in reading would be purely endogenous as the text input does not provide any exogenous temporal rhythm. This would also be in line with the influence of internal linguistic and cognitive factors on saccades during reading (Kliegl et al., 2006; Radach and Kennedy, 2013). In addition to word sampling, we wanted to assess multi-word chunking to substantiate our previous claims on an endogenous character of chunking-related delta-band oscillations (Meyer et al., 2017; Henke and Meyer, 2021). Our measure for this was word-by-word changes in fixation duration because endings of multi-word units are known to show increased reading times (Rayner et al., 2000; Stowe et al., 2018; Tiffin-Richards and Schroeder, 2018). Likewise, chunk endings were accompanied by abrupt changes in reaction time during the learning of visuo-motor sequences (Tosatto et al., 2022). Overall, we expect to see a relationship between temporal regularities in the eye movements and frequency-selective brain activity within the EEG.

## Materials and Methods

### Data acquisition and experimental design

We used the Zurich Cognitive Language Processing Corpus (Hollenstein et al., 2018), which comprises simultaneous eye-tracking and EEG recordings of 12 native English participants (5 female; mean age = 37.5 years; SD = 10.3 years; all right-handed) during reading. We analyzed the natural reading task from the corpus (original task 2) for reasons of analytical simplicity and naturalness (Hollenstein et al., 2018). Participants read 300 sentences and answered a comprehension question after some of them (mean accuracy = 87.96%, SD = 4.93%; for details, see Hollenstein et al., 2018). Sentences were presented in six blocks with recalibration of the eye-tracker in between. For 2 participants, one block is missing, leading to 249 of 250 read sentences (Hollenstein et al., 2018). In the remaining data of all participants, nine sentences had missing eye-tracking data (on average, 0.8 sentences per participant) and were excluded for analysis. Eye movements were recorded with an EyeLink 1000 Plus infrared video-based eye tracker (SR Research) at a sampling rate of 500 Hz (Hollenstein et al., 2018). EEG data were acquired at 500 Hz using a 128-channel EEG Geodesic Hydrocel system (Electrical Geodesics) with Cz as online reference (Hollenstein et al., 2018). Data were bandpass-filtered online from 0.1 to 100 Hz. Further details on the materials and characteristics of the data can be found in the original publication.

### Statistical analysis

#### Eye-tracking analysis

We only considered fixations and saccades that landed on words. The original preprocessing removed fixations not associated with reading (>50 pixels away from any horizontal word position; Hollenstein et al., 2018). On the remaining data of all gaze locations, a Gaussian Mixture Model was trained within each sentence to improve assignment of fixations to text lines. As this procedure was only applied to fixation data in the original preprocessing, we also applied it to saccade landing positions. The Gaussian Mixture Model failed to converge for some sentences (1.9 sentences per participant on average; SD = 2.6); because our main interest here was time rather than space, we kept these for analysis. Gaze positions from single-line sentences were aligned to this line. We removed fixations >1000 ms or <60 ms, which were suggested to reflect technical problems rather than cognitive processing (mean = 2%, SD = 1% of data; see Gagl et al., 2021). Likewise, we removed saccades >80 ms (mean = 3%, SD = 2% of data).

**Figure 1.**
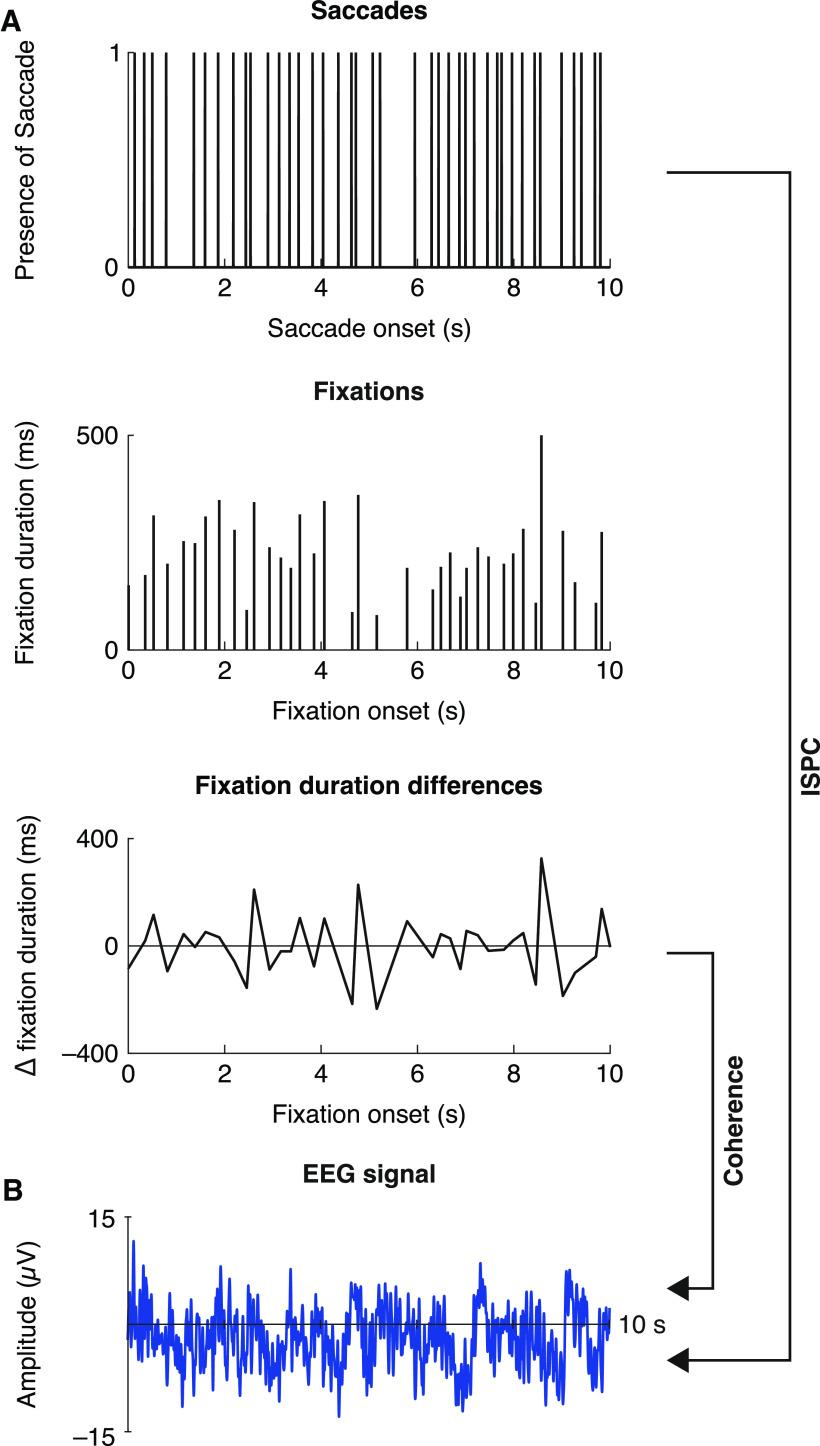
Schematic presentation of the analysis pipeline. ***A***, Eye tracking measures: Binary time series of saccades (1 = saccade onset; 0 = no saccade onset), raw fixation durations, and fixation duration differences (difference between every fixation (*n*) and the fixation directly preceding it; i.e., fixation*_n_* – fixation*_n_*_-1_). ***B***, Phase coherence with EEG data based on ISPC and coherence with the fixation duration differences.

To analyze potential periodicity of eye movements during reading, we converted saccades and fixations into time series (for an overview of this procedure, see Fig. 1). For saccades, we created a binary time series sampled at 1000 Hz set to 1 at saccade onset and 0 at all other time points (Gagl et al., 2021). For fixations, we hypothesized that chunk endings are accompanied by abrupt changes in fixation durations. To highlight these, we first excluded the initial fixation after each backward saccade (= regressions; mean = 21%, SD = 6% of data), which may reflect revision or reinterpretation (Frazier and Rayner, 1982; Schotter et al., 2014) rather than chunking. The remaining fixations had a mean duration of 220 ms (SD = 25 ms). We then computed the difference between every fixation (*n*) and the fixation directly preceding it (i.e., fixation*_n_* – fixation*_n_*_-1_). This outcome was assigned as *y* value to the onset time point of fixation*_n_* as *x* value. The vector was linearly interpolated to a sampling rate of 1000 Hz, effectively resulting in a continuous time series (Fig. 1*A*). Within participant and time series (i.e., separately for the saccade and fixation duration differences time series), we then performed spectral analysis using Welch's PSD. We chose different window lengths to optimize analysis of higher frequencies for saccades and analysis of lower frequencies for fixations; overlap was chosen as half of the window length (window = 4096/8192 ms and overlap = 2048/4096 ms, respectively). Specifically, we followed Gagl et al. (2021) for the saccades yet hypothesized longer intervals for changes in fixation durations. Therefore, we increased the window size to accommodate periodicity within the expected frequency. For statistical analysis within time series across subjects, we compared the mean of the observed spectral variance as an estimate for the effect size against the mean of the spectral variance of a surrogate distribution based on 1000 permutations of each time series. The surrogate distributions for the fixation duration differences were created by shuffling the raw fixation durations while keeping the original fixation time points. Conversion into time series was done according to the observed data. Saccades were permuted by shuffling the binary vector in time. Observed and permuted values were then averaged over participants; statistical significance was met when the observed values exceeded 95% of the permuted values. False discovery rate (FDR) correction was used to control for multiple comparisons at different frequencies. For combined analysis with EEG data, time series were downsampled to 500 Hz.

#### EEG analysis

We used the preprocessing pipeline of the original study by Hollenstein and colleagues (Automagic version 2.6; Pedroni et al., 2019). In brief, we used 104 EEG channels for analysis (excluding the reference electrode), 9 electrooculogram (EOG) channels for the regression of eye movements, and discarded 15 electrodes over the neck and face areas. Bad channels were removed based on a flatline for >5 s, electrode correlation with other channels <0.85, or line noise >4 SDs from the mean across all other channels. Bad channels were interpolated by spherical spline interpolation at the end of preprocessing. Although the data had been high-pass-filtered online, we observed residual drift; therefore, the data were again high-pass filtered at 0.1 Hz (zero-phase 16,500th-order finite impulse response [FIR] filter). To remove line noise, we notch-filtered at 50 ± 3 Hz with a Hamming-windowed-sync FIR filter of 826th order. Eye movements were removed by linearly regressing the EOG channels from the scalp EEG channels. Then, independent component analysis (Makeig et al., 1996) was performed on 1-Hz-filtered (1,650th-order FIR filter) data to facilitate automatic artifact rejection with MARA (Winkler et al., 2011, 2015); components marked as bad were then rejected from the 0.1-Hz-filtered data. Last, data were synchronized with eye movements using EEGLAB's EYE-EEG toolbox (Dimigen et al., 2011), based on shared events across acquisition modalities.

In order to confirm the alignment between the eye movements and the eye-tracking data, we correlated the vertical and horizontal EOGs (average of differential signal of an electrode above and below each eye, and differential signal of the electrodes at the outer canthi, respectively) with the corresponding gaze position (i.e., x-gaze for horizontal EOG and y-gaze for vertical EOG). We removed data where the eye position was not captured by the eye-tracker (∼1% of data). The results indicate a positive relationship between the variables (individual correlation horizontal EOG mean Pearson's *r* = 0.73, SD = 0.23 and vertical EOG mean *r* = 0.28, SD = 0.12; group-level *r*(13,418,532) = 0.59 and 0.22, respectively, both *p* < 0.001), which confirms that the eye-tracker indeed captured the eye movements.

In order to analyze whether periodicity of eye movements during reading is associated with rhythmic neural activity, we investigated phase coherence between these two measures. Statistical analysis was performed using functions from FieldTrip (Oostenveld et al., 2011) and custom MATLAB code (The MathWorks). For saccades, we analyzed the intersaccade phase coherence (ISPC) in the EEG data around saccade onset. We hypothesized ISPC to be increased for frequencies around the saccadic rhythm if this rhythm reflects oscillatory brain activity. We first computed the Fourier transform using Morlet wavelets on the continuous EEG data with frequencies of interest from 0.5 to 10 Hz in 0.5 Hz steps. Then, we segmented the time–frequency data into saccade-locked epochs from ±200 ms (i.e., starting/ending approximately at the previous/subsequent saccade). A few epochs at the beginning and the end of each block were disregarded where the Fourier transform could not estimate low frequencies. We calculated ISPC across remaining epochs and determined statistical significance by comparison to the average ISPC of all nontarget frequencies (Ding et al., 2017) based on 4096 permutations (exhaustive sampling; dependent-samples one-tailed cluster-permutation *t* tests). For the continuous nonbinary time series of fixations, we calculated coherence between the EEG and the fixation duration difference time series computed earlier for the eye-tracking analysis. We epoched the data into sentences and performed a Fourier transform using multitapers from 0.5 to 10 Hz in 0.5 Hz steps with spectral smoothing of 0.5 Hz. We removed sentences with reading durations <2 s to yield sufficiently long windows for analyzing our frequencies of interest (i.e., ≥0.5 Hz; mean = 2%, SD = 3% of data removed). Remaining sentences had a mean reading time of 7.4 s (SD = 2.6 s). Statistical analysis was analogous to the ISPC analysis.

In the next step, we additionally aimed at investigating the relevance of the observed rhythmicity for linguistic processing (see Results). Specifically, we wanted to relate periodic changes in the fixation duration difference time series and their coherence with the EEG to the formation of multi-word chunks. To that end, we performed an additional analysis at sentence boundaries as a proxy of chunk endings (see Fig. 5*A*). We reasoned that, if the observed periodicity reflects the processing of linguistic units, we would expect the phase angles of the fixation duration differences and the EEG to exhibit nonuniformity (i.e., clustering) at sentence boundaries. To assess this, we low-pass filtered the fixation duration differences and the EEG at 2.5 Hz (i.e., isolating the frequency band that showed statistically significant coherence with the fixation duration differences; see Results; two-pass eighth-order Butterworth IIR filter); for the EEG, we selected only the data from the sensor where coherence peaked. We Hilbert-transformed the fixation duration difference time series and the EEG, and extracted analytical phase angles at fixations on the last word of a sentence. We chose fixation onsets because offsets may be confounded with a motor response when switching to the next sentence. Uniformity of phase angles was tested with Rayleigh's test (Berens, 2009). Statistical comparison was performed against the phase-clustering (*z* value) distribution of 1000 surrogate draws of non–sentence-final words to account for the possibility that clustering is related to word onsets as such, rather than sentence-level chunking. Each draw was based on a set of random draws of words that were not sentence-final. For each participant, the number of random draws was equal to the number of observed sentence-final fixations (i.e., extracted angles; mean = 465 angles, SD = 196). Observed and permuted values were averaged over participants, and statistical significance was determined when the observed values exceeded 95% of the permuted values.

## Results

### Eye-tracking results

**Figure 2.**
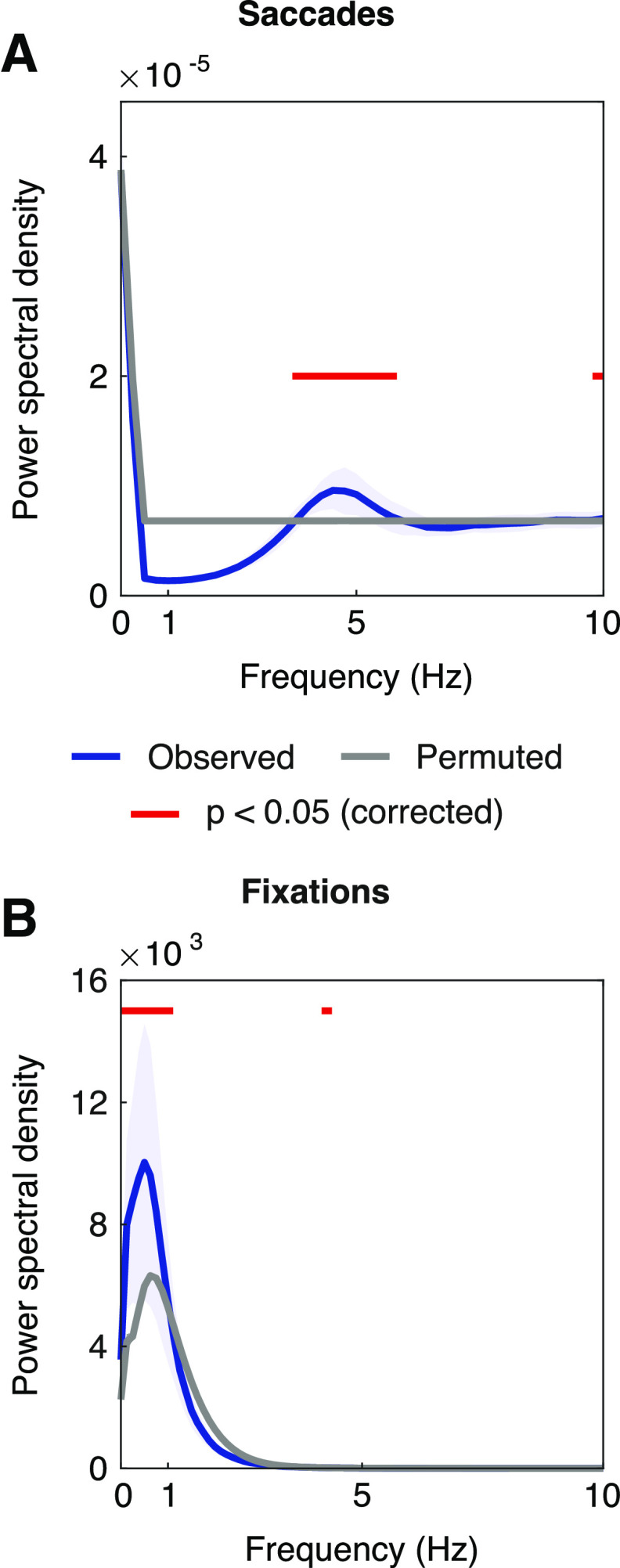
Power spectral density of the eye movements. ***A***, Power spectral density of the binary time series of saccades. ***B***, Power spectral density of the time series of fixation duration differences. ***A***, ***B***, Blue represents observed; gray represents permuted; red represents statistically significant frequencies (*p* < 0.05, FDR-corrected).

Statistical analysis of saccades revealed significant spectral peaks at 4.4 Hz (range 3.7-5.6 Hz) and 10 Hz (*p* < 0.01, FDR-corrected; Fig. 2*A*). Spectral analysis on the fixation duration difference time series showed peaks at 0.49 Hz (range 0-0.98 Hz) and 4.3 Hz (*p* < 0.02, FDR-corrected; Fig. 2*B*).

### EEG results

**Figure 3.**
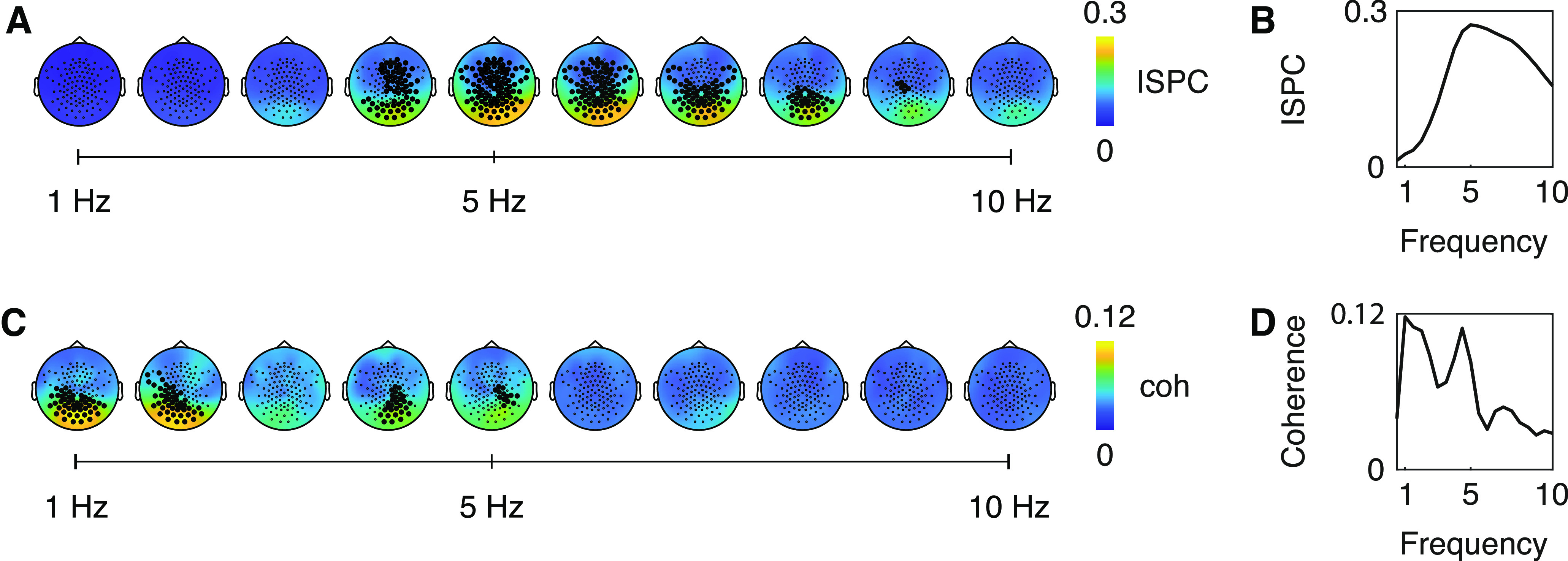
Coherence of eye movements with the EEG data. ***A***, Topographies of ISPC at 130 ms after saccade onset; filled electrodes belong to statistically significant cluster. ***B***, ISPC across frequencies at electrode E83. ***C***, Topographies of coherence between the EEG signal and the fixation duration difference time series on sentences; filled electrodes belong to statistically significant clusters. ***D***, Coherence across frequencies at electrode E83.

**Figure 4.**
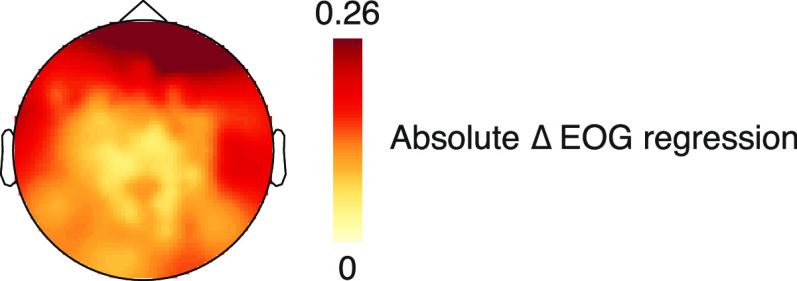
Topography of the removed activity from the EOG regression of the scalp EEG during preprocessing.

ISPC analysis revealed a spatially broad statistically significant cluster with a peak over posterior EEG sensors over the entire time window of ±200 ms around saccade onset within a frequency range from 2.5 to 9.5 Hz (cluster-sum *t*(11) = 528,201, *p* < 0.001; peak coherence at electrode E83 at 5 Hz, 130 ms after saccade onset; Fig. 3*A*,*B*). Coherence analysis on the fixation duration difference time series revealed a significant cluster over posterior EEG sensors from 1 to 2.5 Hz (cluster-sum *t*(11) = 596.91, *p* = 0.011; maximal coherence at electrode E83 at 1 Hz; Fig. 3*C*,*D*) and from 3.5 to 5 Hz (cluster-sum *t*(11) = 334.60, *p* = 0.042; maximal coherence at electrode E90 at 4.5 Hz). As part of our preprocessing, we had regressed the EOG signals from the EEG channels. Given that the EOG regression mainly removed activity from anterior EEG sensors (Fig. 4), the observed coherence over posterior sensors does likely not stem from muscular activity of saccades and fixations alone.

**Figure 5.**
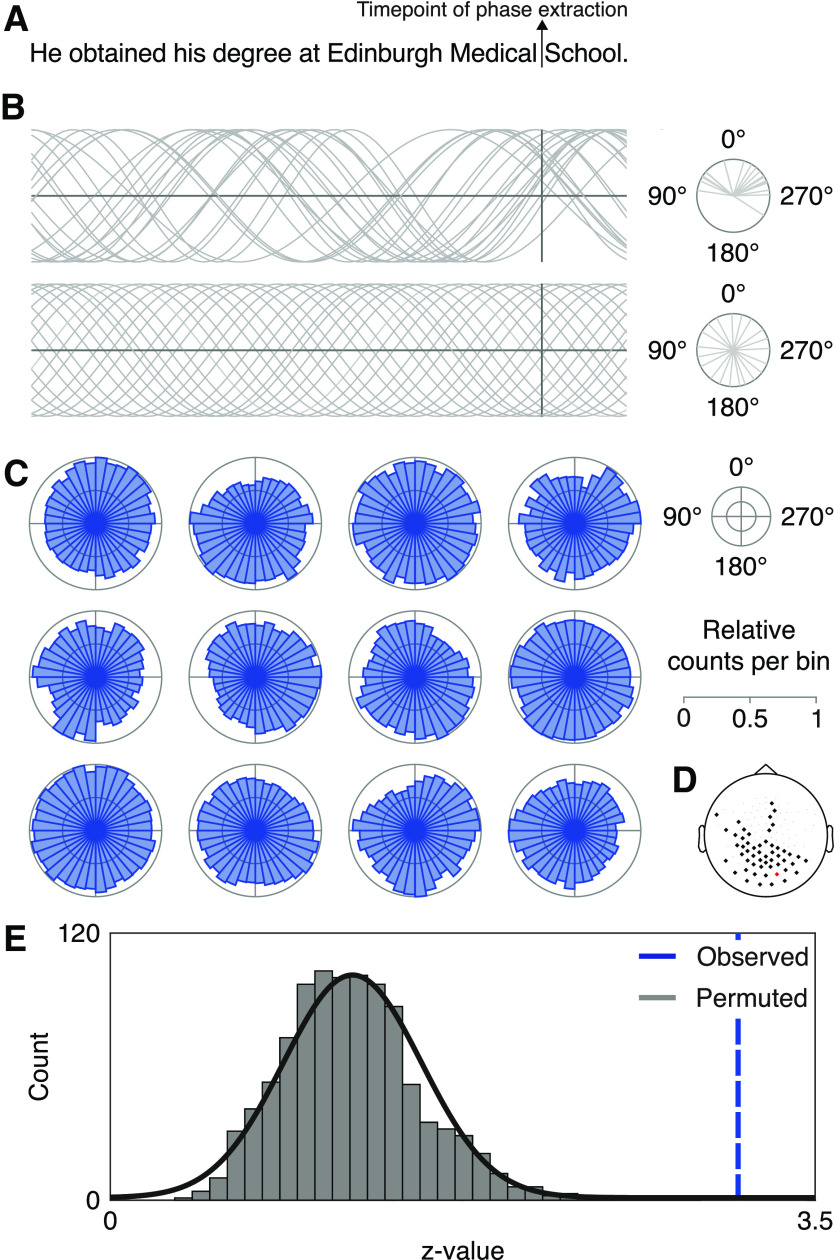
Phase clustering at sentence endings. ***A***, Exemplar sentence with time point of phase extraction (onset of sentence-final word). ***B***, Schematic illustration of a uniform and nonuniform phase-angle distribution at sentence endings. ***C***, Circular histogram of the extracted phase angles from the EEG of all significant electrodes for each participant individually. For illustration, we have normalized the bin count to the maximum of each participant. Additionally, we present all significant electrodes together, although the analysis was conducted for each electrode separately. ***D***, Topography of the significant electrodes (bold), highlighting electrode E83 (red). ***E***, Histogram of the statistical values from the Rayleigh's test of the permuted data (gray) and the statistical value of the observed data (blue) at electrode E83.

To relate our findings of periodic changes in fixation durations as well as their coherence with the EEG to the processing of multi-word units, we analyzed phase clustering at sentence boundaries as a proxy of chunk endings. Phase angles of fixation duration differences at the last word of a sentence did not show significant nonuniformity. This may likely result from the sparse sampling of the data (i.e., one fixation per word), leading to clustering out of randomness in the permuted data. Yet, the distribution of phase angles of the low-pass-filtered EEG at sentence endings at electrode E83 (i.e., the electrode with the maximal coherence with the fixation duration differences) differed significantly from uniformity (*p* < 0.001; mean group-level *z* = 3.12; see Fig. 5*E*). We assessed *post hoc* whether the phase-clustering was restricted to the chosen electrode showing the maximal coherence. To that end, we separately performed the analysis on all electrodes within the significant cluster of coherence at the low frequencies. After FDR correction to account for multiple comparisons over electrodes, 53 of 68 electrodes within the cluster showed statistically significant phase clustering (*p* < 0.05, FDR-corrected; range of mean group-level *z*: 1.67-3.36).

## Discussion

We found that eye movements during naturalistic reading show periodicity that is synchronous with oscillatory activity in the EEG. In two distinct frequency bands, eye movements are synchronized with EEG responses above the visual cortex. Analogous to multiplexed auditory sampling of speech, the (faster) saccadic rhythm may reflect active sampling of words, whereas (slower) rhythmic changes in fixation durations could index an endogenous chunking mechanism that integrates words into larger multi-word units. This interpretation is supported by phase-clustering of the EEG at sensors that show coherence with fixation duration changes. While clustering does not surface in the eye movements as such, this may reflect the challenge of creating an appropriate permutation baseline at the low sampling rate of the fixations. Together, our results could indicate that readers actively align sentence endings to specific phase angles of neural oscillations that subserve linguistic chunking. We also find a peak ∼4 Hz in the fixation duration differences and their coherence with the EEG. This may mirror the saccadic rhythm: Fixation duration differences are inserted at fixation onsets, each following a saccade. Since text does not provide temporal information, rhythmic electrophysiological activity might be an endogenous pacemaker for reading. Our study cannot answer whether this benefits comprehension. This would require a more fine-grained (e.g., word-by-word) assessment of comprehension.

Our findings provide an electrophysiological counterpart to the ∼5 Hz saccade rhythm during reading (Gagl et al., 2021). Saccade frequency and synchronicity with theta-band oscillations converge on a suggested role of theta-band oscillations for auditory processing (Luo and Poeppel, 2007; Peelle et al., 2013; Doelling et al., 2014). Analogous to syllabic sampling of speech, theta-band oscillations may provide optimal sensitivity for processing of words during reading. The theta-band's role in visual attention is well attested (Busch and VanRullen, 2010; Chakravarthi and VanRullen, 2012; Dugué et al., 2015, 2016; Landau et al., 2015; McLelland et al., 2016; Gagl et al., 2021; Michel et al., 2022). During reading, each saccade brings new letters into the focus of attention. Theta-band oscillations have been suggested to modulate saccades, possibly optimizing input gain by proactively increasing neuronal excitability to amplify upcoming stimuli (Maldonado et al., 2008; Rajkai et al., 2008; Melloni et al., 2009; Ito et al., 2011; Hogendoorn, 2016). Saccades may align the times at which information uptake will occur (i.e., fixations) with time points of optimal sensitivity (i.e., a particular phase of the cycle). The pacemaker metaphor is also supported by the finding that the frequency of visual sampling is independent of spatial selection (Re et al., 2019). Although the cognitive process initiating the saccade occurs tens of milliseconds before (e.g., Reichle et al., 2003; Engbert et al., 2005), we certainly cannot claim causality. We thus refrain from interpreting the result as an index of a cognitive process guiding the motor initiation of the saccade.

We also note that our study investigated English, where letter-to-sound associations are nontransparent (for review, see Share, 2008), generally resulting in longer fixations. For instance, a meta-analysis showed that saccadic periodicity ranges from 3.9 to 5.2 Hz across 14 languages (Gagl et al., 2021). This suggests an impact of linguistic processing beyond perceptual sampling. Likewise, saccades during reading are influenced by internal linguistic and cognitive factors (Kliegl et al., 2006; Radach and Kennedy, 2013). Future work should investigate whether the underlying electrophysiological rhythms differ cross-linguistically.

In addition to saccades, we report that changes in fixation duration exhibit periodicity and synchronicity with the EEG within the delta-band. delta-band phase-clustering at sentence endings suggests that readers actively sample larger units at their preferred electrophysiological processing rate. The delta-band serves the active segmentation of speech into multi-word chunks (Ding et al., 2016; Bonhage et al., 2017; Meyer et al., 2017; Jin et al., 2020; Henke and Meyer, 2021). Chunk size may be limited by the wavelength of delta-band oscillations, consistent with a temporal limitation of multi-word units (Vollrath et al., 1992; Roll et al., 2012; Henke and Meyer, 2021). Readers are known to impose implicit segment boundaries to facilitate integration (Fodor and Bever, 1965; Steinhauer and Friederici, 2001; Fodor, 2002; Hirose, 2003; Steinhauer, 2003; Jun and Koike, 2008; Hwang and Steinhauer, 2011). Slowdowns akin to those highlighted by our fixation duration differences have been suggested to reflect this imposition (Hirotani et al., 2006). Yet, by relating delta-band activity to chunking, we do not exclude a more general functionality: Periodic chunking could instantiate a domain-general function in the proactive allocation of attention (Lakatos et al., 2008, 2009). Under this view, the alignment of delta-band cycles to chunks might direct attention to critical information in the stimulus (for an example from audition, see Meyer and Gumbert, 2018). Future research should investigate the behavioral impact on information integration during reading. Moreover, temporal variability makes it unlikely that readers actively sample entire sentences; hence, we require further research to understand the specific type of sampled unit.

Overall, we suggest that during reading, theta- and delta-band oscillations serve active word- and chunk-level visual sampling and integration, respectively. Our results do not suffice to claim causality of endogenous activity for reading. Yet, in evolutionary terms, the practice of reading has certainly developed in the presence of theta- and delta-band oscillations (Knyazev, 2012; Buzsáki et al., 2013). Given the low spatial resolution of EEG, we can only speculate about the specific cortical substrates. All observed effects peaked above the visual cortex, where the relevant rhythms have previously been reported outside of reading (e.g., Park et al., 2016; Bourguignon et al., 2020). We also acknowledge that we cannot conclude that the observed rhythms indeed reflect oscillatory dynamics or rather a sequence of evoked responses. However, note that while saccades and fixations elicit well-investigated evoked components (for review, see Degno and Liversedge, 2020), this would not explain their periodicity, nor temporal regularity of reading. Most importantly, there is no specific periodic change in visual input or motor activity that could act as a counterpart to the chunking rhythm. Future studies should address these limitations.

Our observation of synchronicity between eye movements and the EEG is consistent with the possibility that both frequencies are an endogenous, active means of information selection and structuring (Meyer et al., 2017, 2020a, b; Henke and Meyer, 2021). This might also relate to inner speech produced during reading. On the one hand, reading direct as opposed to indirect speech quotes was associated with increased phase-locking at theta-band frequency (Yao et al., 2021), additionally modulated by a verbal description of speaking rate (e.g., *He said quickly*/*slowly*; Yao and Scheepers, 2011; Stites et al., 2013). This suggests that inner speech is influenced by contextual, meta-cognitive, and/or linguistic factors, similar to saccades during reading (Kliegl et al., 2006; Radach and Kennedy, 2013). On the other hand, it has been suggested that readers generate implicit prosodic contours (for review, see Breen, 2014; also Steinhauer, 2003; Glushko et al., 2022). This may be a mechanism of the speech production system to assist the formation of syntactic structure (Breen, 2014; Drury et al., 2016). It has been argued that the well-known sentence-final wrap-up effect in reading may reflect the insertion of implicit prosodic boundaries (Hirotani et al., 2006), similar to pausing and clause-final lengthening in speech (e.g., Turk and Shattuck-Hufnagel, 2007). Yet, these findings may not necessarily relate to inner speech, but could equally well reflect a chunking process for information integration. For instance, reading involves word skipping and regressive eye movements (Rayner, 1998), suggesting that the linguistic input is sampled in a more sparse way than speech. Sparse sampling may still allow for extracting all necessary information as parafoveal processing allows for accessing a word even before its fixation (for review, see Schotter et al., 2012). Additionally, reading (mostly) samples at one fixation per word, although parafoveal processing can gather additional information. In contrast, speech sampling has often been claimed to occur at the rate of syllables (e.g., Poeppel and Assaneo, 2020), yielding a different amount of linguistic information per neural sample.

During reading, readers extract linguistic information that maps onto speech, suggesting that speech processing is an integral part of reading (Goswami, 2015). Impaired neuronal synchronization to speech has been related to impaired reading (Goswami, 2011). Individuals with developmental dyslexia showed impaired tracking of speech (Molinaro et al., 2016; Power et al., 2016) and nonverbal auditory rhythms (Hämäläinen et al., 2012; Soltész et al., 2013; Lizarazu et al., 2015). This deficit may also underlie reading impairments (Pammer, 2014; Archer et al., 2020). For instance, dyslexic readers display nonrhythmic eye-movement patterns with an increased fixation frequency and longer fixation durations (Lefton et al., 1979; Rayner, 1998; De Luca et al., 1999; Hutzler and Wimmer, 2004; Franzen et al., 2021). A future direction would be to extend our work to reading-impaired populations, also helping to address behavioral benefits of rhythmic reading.

Eye movements during reading are periodic and synchronous with neural oscillations over posterior brain regions. Theta-band oscillations may provide optimal sensitivity for reading single words, whereas slower delta-band oscillations may subserve integration of words into chunks. In this way, neural oscillations endogenously shape reading.
